# Administration of Adipose-Derived Stem Cells Lowers the Initial Levels of IL6 and TNF-Alpha in the Rat Model of Necrotizing Enterocolitis

**DOI:** 10.3390/ijms26146555

**Published:** 2025-07-08

**Authors:** Marek Wolski, Tomasz Ciesielski, Kasper Buczma, Łukasz Fus, Agnieszka Girstun, Joanna Trzcińska-Danielewicz, Agnieszka Cudnoch-Jędrzejewska

**Affiliations:** 1 Department of Pediatric Surgery, Medical University of Warsaw, Zwirki i Wigury 63a, 02-091 Warsaw, Poland; 2 Laboratory of Centre for Preclinical Research, 1st Chair and Department of Experimental and Clinical Physiology, Medical University of Warsaw, Banacha 1B, 02-097 Warsaw, Poland; tomasz.ciesielski@wum.edu.pl (T.C.); kasper.buczma@wum.edu.pl (K.B.); agnieszka.cudnoch-jedrzejewska@wum.edu.pl (A.C.-J.); 3 Department of Pathology, Medical University of Warsaw, Pawinskiego 7, 02-106 Warsaw, Poland; lukaszpiotrfus@gmail.com; 4 Department of Molecular Biology, Institute of Biochemistry, Faculty of Biology, University of Warsaw, Ilji Miecznikowa 1, 02-096 Warsaw, Poland; a.girstun@uw.edu.pl (A.G.); j.trzcinska-da@uw.edu.pl (J.T.-D.)

**Keywords:** necrotizing enterocolitis, adipose-derived stem cells, inflammation mediators

## Abstract

Research in the field of stem cells in necrotizing enterocolitis has primarily focused on the curative role of specific cells—mostly bone marrow and amniotic fluid stem cells. The impact of stem cells on reducing inflammatory cytokine levels in the necrotizing enterocolitis (NEC) model has been studied in accordance with the effects they pose on histopathology. Taking into consideration the possible paracrine mechanism of action of stem cells, our group hypothesized that lowering the initial levels of proinflammatory cytokines may be one of the mechanisms affecting the clinical outcome. A self-modified rat NEC model was used to show the effect of intraperitoneal administration of adipose derived stem cells on the initial levels of interleukin-6 (IL-6) and tumor necrosis factor-alpha (TNF-alfa) in comparison with the interleukin levels in NEC animals and control animals without adipose–derived stem cells (ADSCs) injection. We showed a statistically significant difference in the levels of interleukins when comparing an ADSC injected group and an NEC group. This suggests that one of the mechanisms in which stem cells impact the clinical outcomes in NEC may be by alleviating the initial levels of proinflammatory cytokines.

## 1. Introduction

### 1.1. Necrotizing Enterocolitis (NEC)

Necrotizing enterocolitis is a serious neonatal condition characterized by widespread inflammation and necrosis of the intestinal wall [1]. It predominantly affects pre-term infants, with the risk increasing as birth weight decreases. Infants weighing less than 1000 g are at the highest risk for both morbidity and mortality [2]. The overall incidence of NEC is approximately 1 in every 1000 live births, but this rate can rise to 20% among those with extremely low birth weight. Mortality ranges from 15% to 30% and is inversely related to gestational age and birth weight [2]. Despite being the most common reason for surgical intervention in neonates, the precise pathophysiology of NEC remains unclear. Hypoxia, impaired intestinal perfusion, and microbial colonization are among the primary mechanisms [3]. Major risk factors include prematurity, very low birth weight, early formula feeding, gut dysbiosis, hypoxic events, and congenital cardiac anomalies. Maternal factors—such as infections during pregnancy, metabolic disorders, drug exposure (e.g., cocaine), and perinatal hypoxia—may also contribute to disease onset [2]. Treatment strategies range from conservative management to surgical intervention, with bowel perforation remaining a key surgical indication. Medical treatment typically involves broad-spectrum antibiotics, gastrointestinal decompression, and withholding enteral nutrition. Surgical decisions are guided by the infant’s clinical status and the degree of intestinal damage [2,4]. Among preventive measures, breastfeeding remains the most strongly supported by current evidence [4,5]. Experimental research into NEC utilizes several animal models. Mice and rats are commonly used for evaluating novel therapies, with mouse models offering the advantage of genetic manipulation [5,6,7]. Although more expensive, piglet models provide the most anatomically and physiologically comparable system to the human gastrointestinal tract, making them highly valuable in translational NEC research [6,7].

### 1.2. Cytokines in Necrotizing Enterocolitis

The cytokine signaling pathway plays a central role in the development of necrotizing enterocolitis [8]. The initial mucosal injury, often triggered by hypoxic or hypothermic events, compromises the integrity of the intestinal epithelium. This damage, accompanied by the introduction of formula feeding and colonization by pathogenic bacteria in the underdeveloped gut, promotes the release of pro-inflammatory mediators such as platelet-activating factor (PAF) and tumor necrosis factor-alpha. In addition, bacterial endotoxins like lipopolysaccharide (LPS) disrupt normal mucosal healing by inhibiting the migration of enterocytes, which exacerbates epithelial damage [8,9]. As a result, the mucosal barrier becomes permeable, enabling bacterial translocation and triggering a cascade of inflammatory responses, including vasoconstriction, further immune activation, ischemia, and ultimately necrosis of the intestinal wall. Tissue analyses from NEC patients have shown the elevated mRNA expression of cytokines, including interleukin IL1-beta, IL-8/CXCL8, and TNF [10]. A microarray study conducted by MohanKumar et al. revealed the significantly increased expression of several cytokines in NEC-affected bowels compared with normal tissue, notably IL1-alpha, IL1-beta, IL-6, IL-10, TNF, hepatocyte growth factor (HGF), and vascular endothelial growth factor A (VEGF-A) [10]. Correspondingly, plasma concentrations of IL-6, IL-8/CXCL-8, and IL-10 are elevated in infants diagnosed with NEC, highlighting the systemic inflammatory nature of the disease [10].

Interleukin-6 secretion is significantly enhanced in response to various pro-inflammatory mediators, notably tumor necrosis factor-alpha and interleukin-1 [9,11]. IL-6 plays an important role in the immune response: it stimulates lymphocyte activation, promotes antibody production by B cells, and facilitates the differentiation of cytotoxic T cells [8,9]. During conditions such as systemic inflammatory response syndrome (SIRS) and sepsis, enterocytes increase IL-6 secretion. This cytokine is also produced by intestinal endothelial cells, macrophages, and CD4+ helper T cells [8]. Its expression is further amplified by microbial components like endotoxins, as well as by other cytokines. Additionally, IL-6 is a key inducer of acute-phase proteins, including C-reactive protein (CRP), highlighting its role in systemic inflammation [8].

Tumor necrosis factor-alpha increases the production of IL-1, which acts to promote leukocyte migration, angiogenesis, fever and the acute-phase response [12]. Tumor necrosis factor-alpha is released from macrophages, monocytes, lymphocytes and other cells [13]. In the apoptotic pathway it is associated with the onset of shock with the final effect depending on the receiving receptors [14]. Metalloproteinases (MMP) released from the mucosal mesenchymal cells after TNF-alpha stimulation cause tissue injury and TNF-alpha antibodies have been shown to block this cascade and the subsequent tissue injury. TNF-alpha leads to extracellular matrix breakdown and tissue destruction by specifically upregulating stromelysin 1, MMP-9 and MMP-12 in macrophages, and MMP-19 in epithelial cells [15,16].

### 1.3. Adipose-Derived Stem Cells

Stem cells modulate the immune system primarily through two key mechanisms: direct cell-to-cell interactions and indirect signaling via the secretion of soluble mediators, growth factors, and extracellular vesicles [17]. Immunoregulatory effects arising from direct interactions often occur through paracrine secretion of cytokines, growth factors, and other soluble molecules [17]. Mesenchymal stem cells (MSCs) can be derived from a variety of tissue sources, including the liver, kidney, skin, bone marrow, adipose tissue, placenta, dental pulp, amniotic fluid, amnion, umbilical cord blood, and various fetal tissues [18,19]. Although MSCs from different origins share many biological characteristics, they may vary in aspects such as morphology, immunophenotype, proliferative capacity, differentiation potential, gene expression profiles, proteomic composition, immunomodulatory properties, and their suitability for specific clinical applications [20]. Among these sources, adipose tissue has emerged as a particularly valuable and accessible reservoir of MSCs. Harvesting adipose-derived stem cells poses minimal risk to the donor and typically yields a high number of viable cells [21]. These fibroblast-like cells possess the ability to differentiate into multiple lineages and exhibit surface marker profiles and functional properties that are similar to those of bone marrow-derived stem cells (BMDSCs) [21,22]. Under appropriate conditions, ADSCs are capable of differentiating into cell types from all three germ layers. Notably, one gram of adipose tissue can yield approximately 5 × 10^9^ stem cells, which is about 500 times greater than the MSC yield from the same amount of bone marrow. ADSCs also demonstrate robust proliferation and maintain their multipotent differentiation capacity through multiple cell passages [22,23]. Research has shown that murine ADSCs secrete various angiogenic growth factors, as well as a range of cytokines and chemokines [24]. While data on BMDSCs and amniotic fluid-derived stem cells (AFDSCs) in the context of NEC are limited, studies indicate that their use can significantly reduce the severity and incidence of NEC in animal models [25,26,27]. In our previous research we have shown that administration of ADSCs before the onset of the disease lowers the levels of inflammatory cytokines IL-1 and IL-6 in the rat model of NEC [28].

The hypothesis of the study was that intraperitoneal administration of adipose-derived stem cells would affect the initial levels of proinflammatory cytokines IL-6 and TNF-alpha in model NEC animals. The rationale behind this assumption was the previous observation of our group that ADSC administration diminishes the levels of proinflammatory cytokines in the NEC model. Taking into consideration the results of our previous work [28,29] and publications, which suggest that the effect of stem cells does not require engraftment but substantially relies on the paracrine effect of those cells [30], as well as the suggested mechanism of the hyperinflammatory state in post-NEC patients [31], we assumed that ADSCs administered before subjecting model animals to NEC-causing factors would lower the cytokine levels and therefore attenuate the inflammatory state before the outbreak of the disease.

## 2. Results

### 2.1. Engraftment

To assess the level of engraftment into the bowel tissue, injected human adipose-derived stem cells were stained with anti-CD90 antibodies using the streptavidin–biotin method.

**Figure 1:**
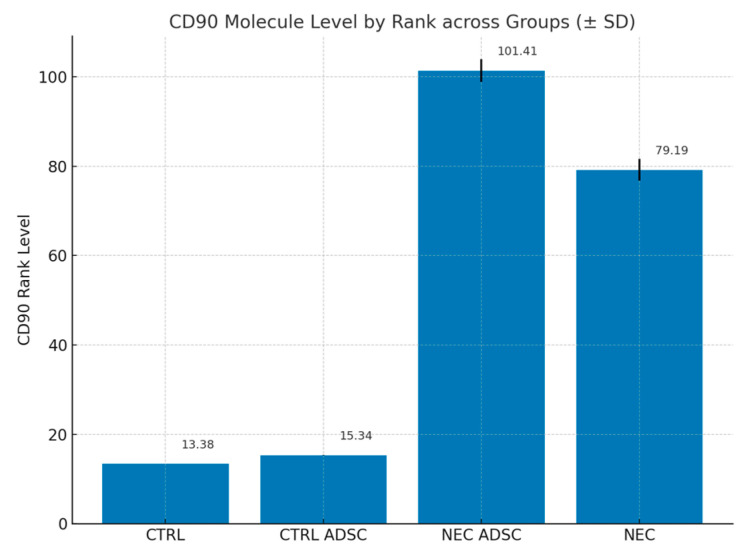
Levels of CD90 antibodies—expressed by rank across groups. Standard Deviation marked with black lines.

The results of CD90 antibody levels in all groups are presented in the table and figure below (Table 1, Figure 1).

### 2.2. Histopathology

For histopathological analysis, each specimen (n = 143) was preserved in a 10% neutral buffered formalin solution. The fixed tissues were embedded in paraffin, sectioned into 3 µm slices, and routinely stained with hematoxylin and eosin (H&E) for morphological assessment. Subsequently, they were examined at higher magnifications (200× and 400×) and scored semi-quantitatively using a four-tier grading system: grade 0—no change in the bowel wall; grade 1—partial villous atrophy; grade 2—epithelium sloughing and/or necrosis in the upper part of the atrophic villi; and grade 3—total loss of villi, necrosis of the intestinal wall [5,28].

**Figure 2:**
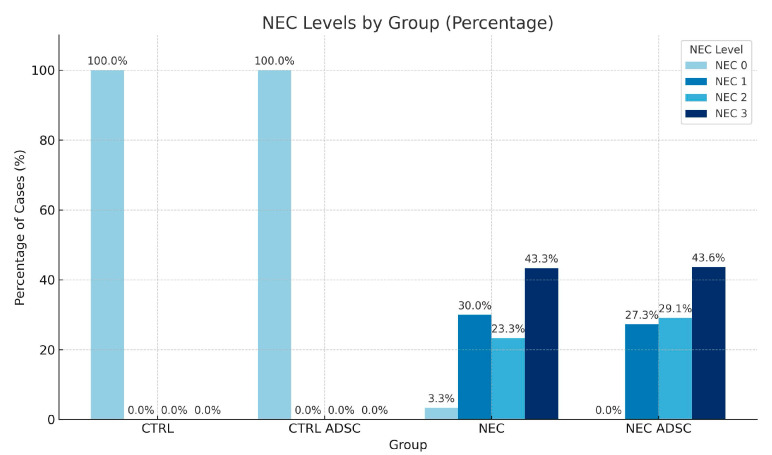
Histopathological results. Percentage of each severity grade by group.

The results of the histopathological analysis of all groups are presented in the table and figure below (Table 2, Figure 2).

The association between NEC occurrence and the experimental groups NEC and NEC ADSC was evaluated using the chi-square test. Due to the presence of expected values below 5, Fisher’s exact test was employed to ensure accurate significance estimation. The results revealed no statistically significant relationship between NEC incidence and group assignment [χ^2^(6) = 4.36; *p* = 0.628]. The distribution of NEC cases appeared comparable across all experimental groups. A visual representation of these findings is provided in Figure 2.

### 2.3. Cytokine Concentrations

Levels of IL-6 and TNF-alpha in CTRL, CTRL-ADSC, NEC and NEC-ADSC groups were assessed with commercial ELISA kits. The results are presented in the tables below (Table 3 and Table 4).

**Figure 3:**
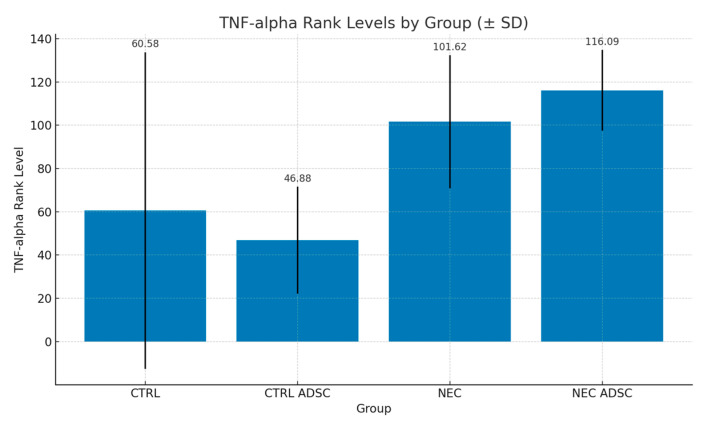
TNF-alpha rank levels within groups (pg/mL). Standard Deviation marked with black lines.

**Figure 4:**
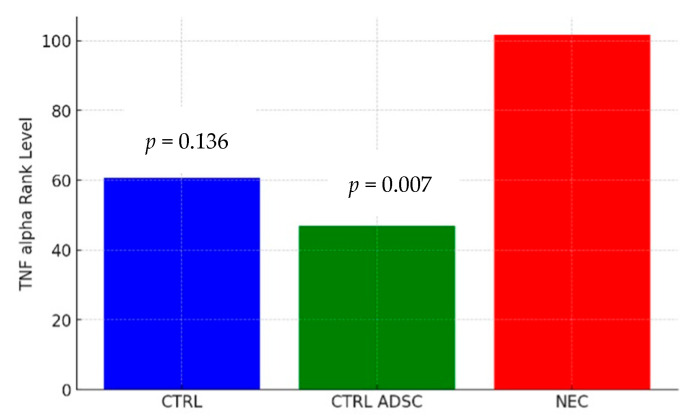
TNF-alpha rank levels within CTRL and CTRL ADSC groups compared with NEC group (pg/mL).

**Figure 5:**
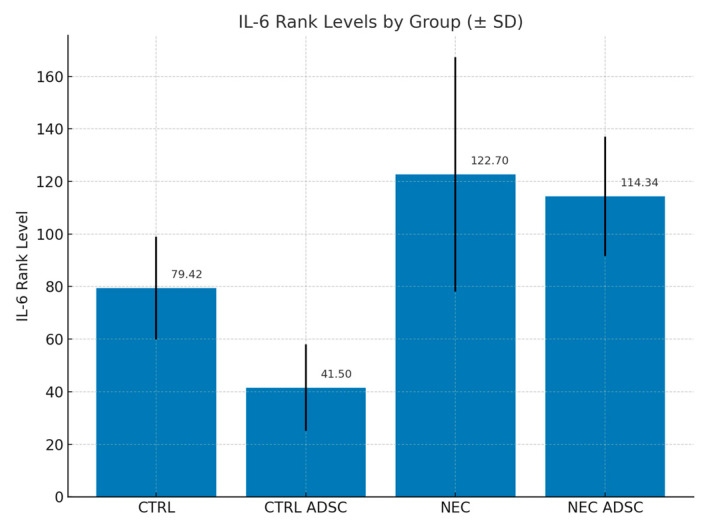
IL-6 rank levels within groups (pg/mL). Standard Deviation marked with black lines.

**Figure 6:**
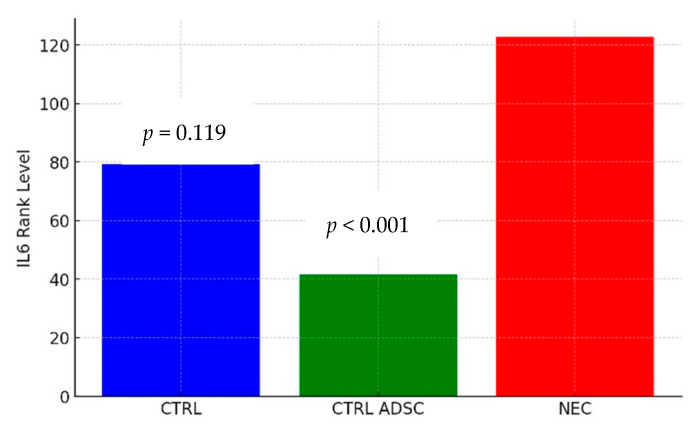
IL-6 rank levels within CTRL and CTRL ADSC groups compared with NEC group (pg/mL).

The difference in TNF-alpha levels between the CTRL and NEC groups was not statistically significant (*p* = 0.136). The average severity of TNF-alpha values in the CTRL group was statistically similar to that in the NEC group, with corresponding mean ranks of 60.58 and 101.62, respectively. The difference in TNF-alpha levels between the CTRL ADSC and NEC groups was statistically significant (*p* = 0.007). The average severity of TNF-alpha values in the CTRL ADSC group was significantly lower compared with the NEC group, with corresponding mean ranks of 46.88 and 01.62, respectively. The difference between the NEC ADSC and NEC groups was not statistically significant (*p* = 0.748). The average intensity of TNF-alpha variable levels in the NEC ADSC group was statistically similar to those observed in the NEC group, with rank means of 116.09 and 101.62, respectively. The difference in IL-6 levels between the CTRL and NEC groups was not statistically significant (*p* = 0.119). The average severity of IL-6 values in the CTRL group was statistically similar to those observed in the NEC group, with corresponding mean ranks of 79.42 and 122.7, respectively. The difference between the CTRL ADSC and NEC groups was statistically significant (*p* < 0.001). The average severity of IL-6 values in the CTRL ADSC group was significantly lower compared with the NEC group, with corresponding mean ranks of 41.5 and 122.7, respectively. The difference between the NEC ADSC and NEC groups was not statistically significant (*p* = 0.892). The average intensity of IL-6 variable levels in the NEC ADSC group was statistically similar to those observed in the NEC group, with mean ranks of 114.34 and 122.7, respectively. The differences between groups are presented in the figures below (Figure 3, Figure 4, Figure 5 and Figure 6).

## 3. Discussion

Necrotizing Enterocolitis is a general inflammation state that results in bowel necrosis and death. Understanding, from our previous research [28], that administration of ADSCs before the onset of the disease alleviates the levels of inflammatory cytokines in NEC but exerts a much lesser effect when the stem cells are injected during the course of the disease [29], we sought to look for the potential mechanism of this discrepancy and hypothesized that stem cells will affect the initial levels of inflammatory cytokines and therefore could elevate the prophylactic potential in this mechanism. For this reason, we utilized the hypoxia–hypothermia formula feeding rat NEC model and focused mainly on the initial levels of proinflammatory cytokines IL-6 and TNF-alpha by comparing the NEC group to animals not subjected to the NEC protocol and injected with ADSCs and healthy control animals. We also analyzed the effect the ADSCs had on NEC animals considering the cytokine levels.

The inflammatory response induced by necrotizing enterocolitis results in the elevation of the levels of all major inflammatory cytokines involved in NEC [8,32]. Benkoe et al. have shown a strong increase in serum levels of IL-6, IL-8 and IL-10 in clinical NEC patients. In the historical work by Markel et al., the characteristics of major cytokines involved in NEC, like IL-1, IL-6, TNF-alpha and others, are described. Mesenchymal stem cells, mostly by their paracrine effects, have the ability to modulate inflammatory response and lower the levels of inflammatory cytokines [33,34]. On the histopathological level this was shown by McCulloch et al., who observed a reduction in histopathological NEC across model groups with different types of stem cells [25,26]. In the works of Zani et al., the pattern of effectiveness in limiting necrotizing enterocolitis by integrating into the intestinal wall, limiting apoptosis and enhancing proliferation was demonstrated for amniotic fluid stem cells [35]. Our group has shown that adipose-derived stem cells are also effective in alleviating the levels of inflammatory cytokines in the model of NEC [28]. Mesenchymal cells found in the adipose tissue are a subset of stem cells that do not pose the typical ethical dilemmas and can be easily harvested with liposuction techniques. They do not differ from other MSCs as far as immunomodulatory, anti-inflammatory and pro-angiogenic properties are concerned [17,36]. Apart from the above cited work by our group, the only research concerning adipose tissue in the setting of necrotizing enterocolitis is the work by Mimatsu et al. [37]. This group showed that differentiated adipocytes, and not ADSCs, diminish mortality and alleviate intestinal healing in NEC by modulating fatty acid-related protein and diminishing inflammation, including IL-1 and IL-6 levels [37]. In the present study, we have demonstrated that ADSCs influence the initial levels of inflammatory cytokines; however, this does not translate into differences in the final histopathological or biochemical profile of the disease. These findings highlight the potential of stem cells to mitigate the inflammatory state in tissues. On the other hand, the experimental model proved ineffective in capturing clinical differences, which may be attributed either to the intensity of the stimuli used to induce NEC or to the arbitrarily selected timing of ADSC administration.

Liu et al. have shown that the initial expression of TLRs and cytokines precedes histological injury in experimental NEC [38]. In the work of Snyder et al. [31], the authors aim to prove that the dysregulation of the inflammatory system in NEC makes the “NEC recovery” patients more vulnerable to disease-inducing factors in the future. They show that enteroids generated from patients that have recovered from necrotizing enterocolitis have an alleviated inflammatory response when exposed to NEC-causing factors then the controls [31]. This hyperinflammatory state may play a significant role in the “second hit” of NEC stimuli but also draws attention to the initial potential of the inflammatory system in response to NEC stimuli. In our opinion the ability of ADSCs to lower the inflammatory potential could affect the inflammatory cascade and attenuate the inflammatory response, therefore limiting the histopathological and clinical picture of NEC. Pisano et al., in their work, have focused on the possible mechanisms of this inflammation-lowering potential [39]. The group tried to identify the role of stem cells, heparin-binding epidermal growth factor-like growth factor, and stem cell-derived exosomes in NEC prophylaxis [39]. The hypothesis was that the main mechanism behind the prevention of the disease is not engraftment of stem cells into the bowel tissue but paracrine or endocrine secretion of factors, exosomes, or vesicles [39]. This is in line with our results that revealed the effect of stem cells on reducing inflammatory cytokines levels before the onset of the model with little potential to engraft into the not-yet-inflamed bowel tissue. The engraftment of the cells as shown by the levels of CD90 molecule was much higher in the NEC-affected bowel fragments, which can be explained by the level of inflammation in these regions, a level that promotes migration. The elevated levels of CD90 molecule in the NEC group without the addition of ADSCs can be explained by the background signal caused by inflamed or necrotic intestinal tissue.

In our previous works we have shown the difference in ADSCs effectiveness in alleviating the inflammatory cytokines IL-6 and IL-1 levels depending on the time when the stem cells are injected into the model of NEC [28,29]. The stem cells exerted a higher capacity to affect the inflammation when injected as a prophylactic than as a curative agent. Following Snyder’s idea of hyperinflammatory state in NEC recovery patients [31], the results of our studies could suggest that the actual inflammatory state of the patient may play a crucial role in the development of the clinical picture of necrotizing enterocolitis and the trajectory of the disease. The clinical picture of NEC, and especially the factors affecting its most devastating fulminant form, remains unpredictable regardless of the decades of research. Authors have focused on the rapid course of the disease, characterized by urgent onset and prompt deterioration and usually resulting in death, with a lack of unique characteristics [40]. In their work from 2021, Garg et al. conclude that fulminant NEC patients frequently developed thrombocytopenia, lymphopenia, neutropenia, and leukopenia or received RBC transfusions after, or platelet transfusions before, the onset of NEC [41]. This observation could overlap with the discussion on the hyperinflammatory state and potentialization of inflammation in this group of patients, a group for whom a properly timed prophylactic intervention aimed at lowering the inflammation potential could be of high benefit. The only clinical application of stem cells to date has been reported by Akduman et al. [42]. Following the transfusion of 1 × 10^7^ allogeneic umbilical cord mesenchymal stem cells (UCMSCs) in a post-colectomy NEC patient, the authors observed a favorable clinical outcome without any adverse effects. Although the conclusions are limited by the nature of a single case report, these findings might suggest the hypothesis that intravenous administration of allogeneic or autologous ADSCs could in future represent a potential prophylactic strategy for patients at risk of developing NEC. This notion is further supported by evidence that small volumes of adipose tissue can yield sufficient numbers of viable stem cells, which retain their functional properties through at least five passages, as demonstrated by Ren et al. [22].

The limitations of the study include the limited amount of specific cytokines studied and the comparison with other stem cell types. In future research, we believe it is worth enlarging the groups to better elucidate the statistical differences. Additionally, the perspective of testing other stem cell types and their ability to influence the initial inflammatory state of the NEC model animals could be of high importance, as we believe it is one of the main mechanisms in which the stem cells attenuate the clinical picture of NEC. Although the primary objective of the study was to compare animals unaffected by NEC with a pure NEC group, the inability of our model to reveal changes in the histopathological and biochemical profile of NEC following ADSC administration represents a limitation. This issue could potentially be addressed by adjusting the timing of stem cell administration or modifying the NEC-inducing stimuli. Employing a model previously shown to effectively demonstrate histopathological differences in NEC with other types of stem cells, like, for example, in the works of McCulloh et al. [26], may offer further insight into the effects of ADSCs in this context. Additionally, due to technical constraints, we did not assess plasma levels of proinflammatory cytokines—an omission that may be important given the potential future clinical application of ADSCs.

## 4. Materials and Methods

The experiment was approved by the local ethical committee for animal experiments (WAW/093/2021 from 16 June 2021, amended 20 July 2022). To present the effect of ADSC administration on the initial levels of inflammatory cytokines IL-6 and TNF-alpha, we utilized a self-modified hypoxia–hypothermia formula feeding rat model of necrotizing enterocolitis [5]. This model has been previously used by our group to present the effect of ADSCs on prophylaxis and treatment of NEC [28,29].

A total number of 143 newborn SPRD/Mol/Lodz rats were used in the experiment (n = 143). Sixteen rats in the CTRL ADSC group (no NEC protocol, ADSCs added) were subjected to intraperitoneal injection of 6 × 10^5^ labeled cells in 50 uL PBS, each 24 h after birth. Twelve animals were the control group (no NEC protocol, no ADSCs added), 60 rat pups in the NEC group were injected with 50 μL of PBS and had undergone the NEC procedure, and 55 animals in the NEC ADSC group (NEC protocol, ADSCs added) were subjected to intraperitoneal injection of 6 × 10^5^ labeled cells in 50 μL PBS, each after 24 h of the protocol duration. No inclusion or exclusion criteria were set, and animals were assigned into groups by litter. No animals were excluded from the analysis. Only one investigator was aware of the allocation. All measurements and analyses were conducted by investigators on blinded data. The samples for all analyses were collected after 72 h of protocol duration, or before in case of spontaneous death.

The sample size needed to demonstrate that the effect of the study was calculated based on the results of the pilot study and the literature [5,28,29]. To demonstrate statistical significance using the chi-square test, with a significance level of 0.05, a test power of 70% and a dropout rate of around 50%, the required number of animals in groups undergoing NEC protocol is 60 and is 10 in groups used for control or with no NEC protocol used. All animals were kept under the same conditions in individual ventilated cages.

### 4.1. NEC Protocol

The protocol consisted of exposure to 100% nitrogen atmosphere for 60 s in individual ventilated cages for hypoxia. The hypothermia was acquired by exposing the animals to 10 min. of a temperature of 4 degrees Celsius. Animals were fed with a commercial formula milk by a 0.5 mm pipette. The animals were euthanized after a set time of 72 h. and the peritoneal fluid and bowel samples were obtained.

### 4.2. Adipose-Derived Stem Cells

**Figure 7:**
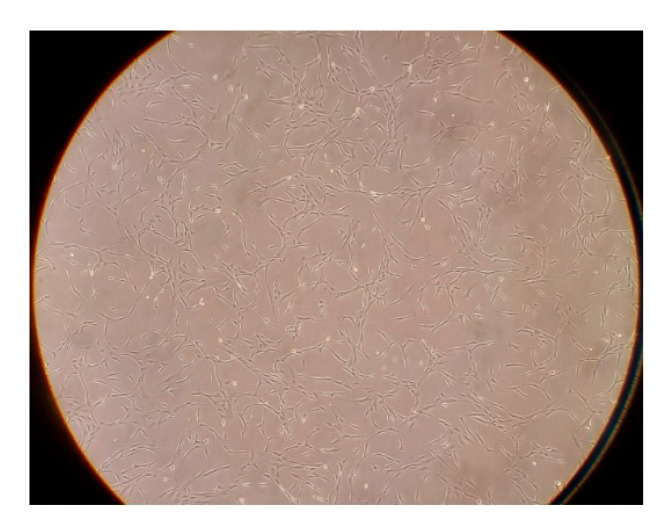
Stem cell culture. Microscope Olympus ck30, WIK10x/20L Magnification: 40× Source: own materials.

StemPro human adipose-derived stem cells (Gibco, Thermo Fisher Scientific, Life Sciences Solutions, #R7788110, Carlsbad, CA, USA) were cultured in MesenPRO RS Basal Medium supplemented with MesenPRO RS Growth Supplement (included with the ADSC cells by Gibco), 2 mM L-glutamine, 100 μg/mL streptomycin, and 100 U/mL penicillin. The cells were cultured at 37 °C in a humidified environment containing 5% CO_2_ (Figure 7). Cells at passage levels below 5 were selected. After reaching 80% confluency, the cells were washed with PBS and detached from the culture vessels with Accutase solution. The cells were collected, centrifuged at 250× *g* for 5 min at room temperature, counted, washed with PBS, and centrifuged again. Finally, the cells were resuspended in PBS and portioned 6 × 10^5^ cells per 50 μL.

### 4.3. Engraftment Analysis

**Figure 8:**
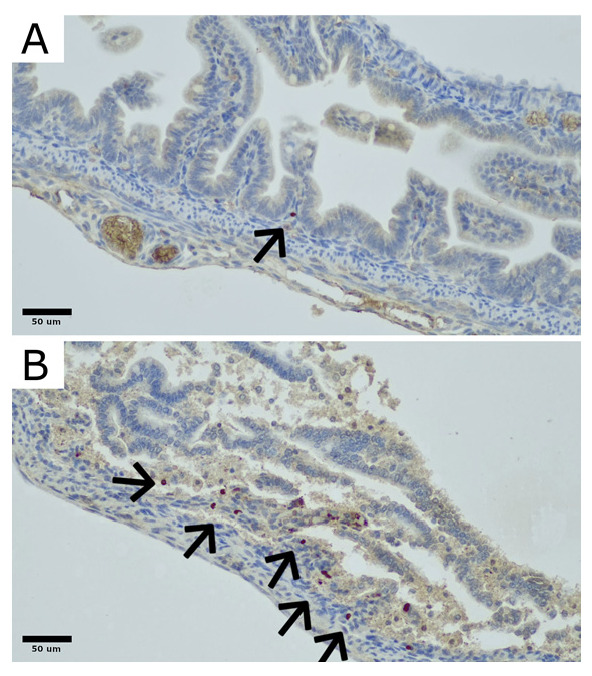
Examples of CD90-positive cell distribution in the rat bowel: (**A**) Section of colon showing partial villous atrophy with only a single CD90-positive cell (indicated by an arrow). (**B**) Section of colon exhibiting sloughing and necrosis of the upper parts of atrophic villi, with scattered CD90-positive cells present in the mucosa and submucosa (indicated by arrows). Original magnification: 400×. Microphotographs were captured using the OPTIKA LITEView software (OPTIKA, Version: Windows x64 2.1.24744.20240303; OPTIKA, Ponteranica, Italy). Source: author’s materials.

To identify the CD90 cells, samples of rat bowel were stained with an anti-CD90 polyclonal rabbit antibody (LifeSpan Biosciences, Newark, CA, USA) according to the manufacturer’s guidelines (Figure 8). Regions with highest density of CD90-positive cells were identified at 100× magnification. In these selected regions, the number and distribution of CD90-positive cells were quantitatively analyzed at 200× magnification.

### 4.4. Cytokine Concentrations—ELISA Analysis

TNF-alpha and IL6 levels in peritoneal fluid were measured with a commercial ELISA test kit provided by R&D Systems, Minneapolis, MN, USA, per the manufacturer’s instructions. The procedure began with adding 50 μL of assay diluent to each well, followed by 50 μL of standard, control, or sample. After gentle mixing, the plate was covered and incubated for 2 h at room temperature. After incubation, the wells were aspirated and washed five times with wash buffer. Then, 100 μL of IL-6 or TNF-alpha conjugate was added to each well, followed by 2-h incubation at room temperature. After a second wash, 100 μL of substrate solution was added, and the plate was incubated for 30 min, protected from light. Finally, 100 μL of stop solution was added, and the optical density was measured at 450 nm within 30 min, with wavelength correction applied at 540. The measurement was repeated three times for each sample.

### 4.5. Histopathology

**Figure 9:**
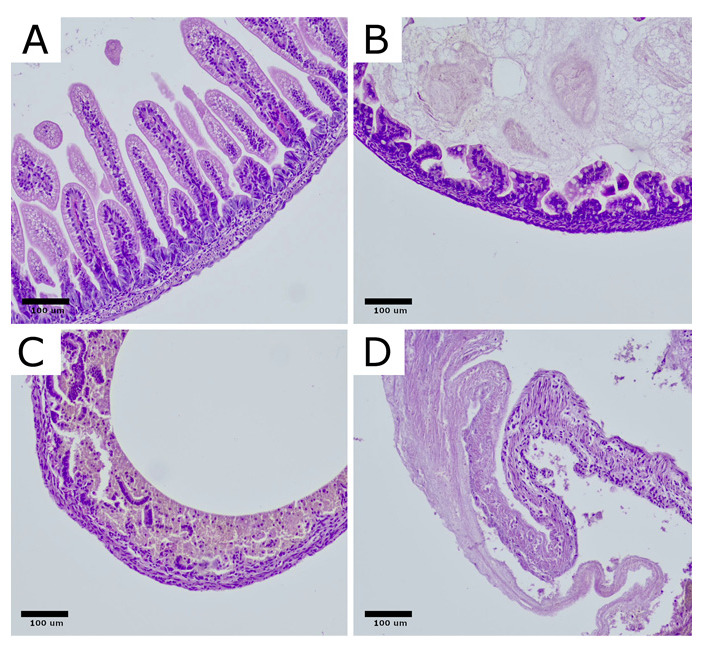
Histological changes in intestinal architecture of rats with NEC. Rat bowel stained with H&E showing representative sections for each morphological severity score. (**A**) normal ileum, NEC score 0. (**B**) NEC score 1, partial villous atrophy. (**C**) NEC score 2, sloughing and/or necrosis of upper parts of atrophic villi, (**D**) NEC score 3, total loss of villi and necrosis of the intestinal wall. Original magnification 200×. Microphotographs were captured using OPTIKA LITEView software (OPTIKA, Italy). Source: own materials.

After fixation in a 10% neutral buffered formalin solution, samples were embedded in paraffin, sectioned at 3 µm thickness, and stained with hematoxylin and eosin (H&E) for morphological assessment. Initially, slides were examined at a low magnification (40× scanning) to assess the general structure of the rat bowel. Subsequently, slides were viewed at 200× and 400× magnifications and evaluated semi-quantitatively using a four-tier NEC severity grading scale: grade 0—no change in the bowel wall; grade 1—partial villous atrophy, grade 2—epithelium sloughing and/or necrosis in upper part of atrophic villi, grade 3—total loss of villi, necrosis of the intestinal wall [4,41]. (Figure 9).

### 4.6. Statistical Analysis

The analyses were conducted using R software (R Core Team, 2023). Visualization of the results was performed using the “ggplot2” graphics package (Wickham, 2016) and ChatGPT 4.0 by OpenAI. To assess the effect of the group variable on TNF-alpha, IL6 and CD90, a one-way analysis of comparisons for independent samples was conducted. Given the non-parametric nature of the data, the Kruskal–Wallis test was applied.

## 5. Conclusions

Administration of adipose-derived stem cells before the onset of the disease lowers the initial levels of IL-6 and TNF-alpha in the rat model of necrotizing enterocolitis.

This may be the potential mechanism of the impact of stem cells on the clinical picture of necrotizing enterocolitis but further research is necessary possibly with the use of a better tailored model.

## Figures and Tables

**Table 1:** Levels of CD90 antibodies—expressed as percentage of the total cells present in the bowel mucosa and submucosa.

Group	n	Min	Max	M	SD	Rank
CTRL	12	0	10	0	0	13.38
CTRL ADSC	16	0	10	0.02	0.07	15.34
NEC ADSC	55	0	10	3.1	2.58	101.41
NEC	60	0	10	2.17	2.4	79.19

**Table 2:** Histopathological results. Number of samples in each severity grade.

Group	CTRL	CTRL ADSC	NEC	NEC ADSC
**NEC**				
0	12 (100%)	16 (100%)	2 (3.33%)	0 (0.00%)
1	0 (0%)	0 (0%)	18 (30.00%)	15 (27.27%)
2	0 (0%)	0 (0%)	14 (23.33%)	16 (29.09%)
3	0 (0%)	0 (0%)	26 (43.33%)	24 (43.64%)
**Total**	12 (100%)	16 (100%)	60 (100.00%)	55 (100%)

**Table 3:** Levels of TNF-alpha within groups (pg/mL).

Group	n	Min	Max	M	SD	Rank
CTRL	12	35.33	300.4	71.14	73.16	60.58
CTRL ADSC	16	6.45	100.3	46.26	24.73	46.88
NEC	60	23.15	212.4	71.22	30.78	101.62
NEC ADSC	55	28.5	109.4	75.78	18.68	116.09

**Table 4:** Levels of IL-6 within groups (pg/mL).

Group	n	Min	Max	M	SD	Rank
CTRL	12	35.33	100.36	67.75	19.60	79.42
CTRL ADSC	16	25.44	85.46	51.54	16.45	41.50
NEC	60	25.30	220.47	94.55	44.64	122.70
NEC ADSC	55	29.46	150.38	81.12	22.7	114.34

## Data Availability

All data are available on request from the authors.
